# Identification of a novel small-molecule Keap1–Nrf2 PPI inhibitor with cytoprotective effects on LPS-induced cardiomyopathy

**DOI:** 10.1080/14756366.2018.1461856

**Published:** 2018-04-25

**Authors:** Cheng-Shi Jiang, Chun-Lin Zhuang, Kongkai Zhu, Juan Zhang, Luis Alexandre Muehlmann, João Paulo Figueiró Longo, Ricardo Bentes Azevedo, Wen Zhang, Ning Meng, Hua Zhang

**Affiliations:** a School of Biological Science and Technology, University of Jinan, Jinan, China;; b School of Pharmacy, Second Military Medical University, Shanghai, China;; c Faculty of Ceilandia, University of Brasília, Brasilia, Brazil;; d Institute of Biological Sciences, University of Brasília, Brasilia, Brazil

**Keywords:** Keap1, Nrf2, protein–protein interaction inhibitor, cytoprotective, septic cardiomyopathy

## Abstract

A new Keap1–Nrf2 protein–protein interaction (PPI) inhibitor **ZJ01** was identified from our compound library by fluorescence polarization assay, surface plasmon resonance, molecular docking and molecular dynamics simulation. **ZJ01** could *in vitro* trigger Nrf2 nuclear translocation, subsequently resulting in increased mRNA levels of Nrf2 target genes HO-1 and NQO1. Meanwhile, **ZJ01** suppressed LPS-induced production of ROS and the mRNA levels of pro-inflammatory cytokines TNF-α, IL-1β and IL-6 in H9c2 cardiac cells. Moreover, in an *in vivo* mouse model of septic cardiomyopathy induced by intraperitoneal injection of lipopolysaccharide, **ZJ01** demonstrated a cytoprotective effect, upregulated Nrf2 protein nuclear accumulation, and remarkably suppressed the abovementioned cytokine levels in cardiomyocytes. The results presented herein provided a novel chemotype for the development of direct Keap1–Nrf2 PPI inhibitors and suggested that compound **ZJ01** is a promising drug lead for septic cardiomyopathy treatment.

**ZJ01** was identified as a new Keap1–Nrf2 PPI inhibitor and drug lead for septic cardiomyopathy treatment by *in vitro* and *in vivo* experiments.

## Graphical abstract

**Figure:**
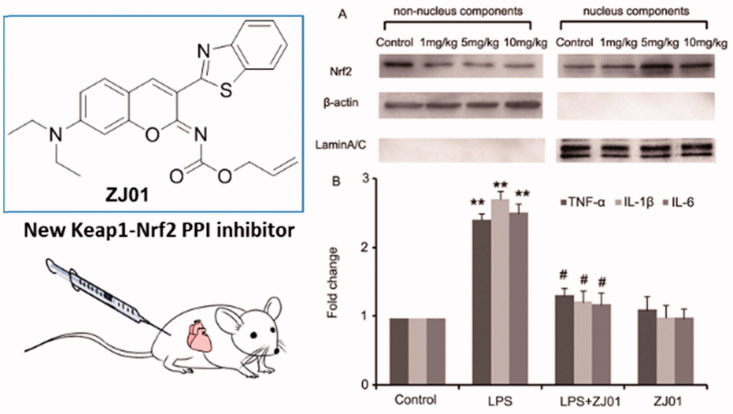

## Introduction

Sepsis, a major cause of death worldwide, is a systemic inflammatory syndrome caused by serious infections^1^. According to the statistics, up to 70% of patients with sepsis are affected by septic cardiomyopathy, and the dysfunction of cardiomyocytes contributes to a high mortality rate^2^. Currently, septic cardiomyopathy remains a great challenge for clinical treatment. Lipopolysaccharide (LPS), the major component of the outer membrane of gram-negative bacteria, has proven to be a key stimulator of sepsis^3^. Under septic conditions, LPS can evoke an inflammatory response in innate immune cells, as well as non-immune cells (e.g. cardiomyocytes) expressing LPS-pattern recognition receptors^4^. More importantly, cardiac dysfunction frequently occurs in patients with sepsis and animals injected with LPS^5^ ^,^ ^6^. Increasing evidence indicates that control of the oxidative stress and inflammatory response in cardiomyocytes could alleviate LPS-induced cardiac injury^7–9^.

It has been known that nuclear factor erythroid 2-related factor 2 (Nrf2) can protect cells against oxidative stress and repress inflammation^10^ ^,^ ^11^. In the anti-oxidant defense system, the expression of a number of key genes, such as NAD(P)H: quinone oxidoreductase 1 (NQO1), heme oxygenase-1 (HO-1), superoxide dismutase (SOD) and glutathione S-transferase (GST)^12^, is mainly regulated by three components: Nrf2, Kelch-like ECH-associated protein 1 (Keap1) and anti-oxidant response elements (ARE)^13^. Nrf2 activity is regulated mainly by Keap1^14^. Under unstressed conditions, Nrf2 is negatively regulated by Keap1 via proteasomal degradation to remain at low cellular concentrations. Upon oxidative stress, reactive oxygen species (ROS) and/or electrophilic agents modify the reactive cysteine residues in Keap1, resulting in conformational changes and deactivation of Keap1. Subsequently, Nrf2 is activated after escaping from Keap1-mediated degradation and then translocate into the nucleus where it induces the transcription of the aforementioned downstream genes by binding to ARE^14^. More interestingly, a recent study demonstrated that Nrf2 could inhibit pro-inflammatory cytokine transcription through direct DNA binding^15^. Interestingly, other studies have highlighted the important role of Nrf2 activation in protecting cardiomyocytes from LPS-induced injuries^16^. Thus, to activate Nrf2 via disrupting the Keap1–Nrf2 protein–protein interaction (PPI) represents an attractive strategy to develop drugs for diseases involving oxidative stress and inflammation, such as cardiomyopathy^16^ ^,^ ^17^.

**Figure 1.**
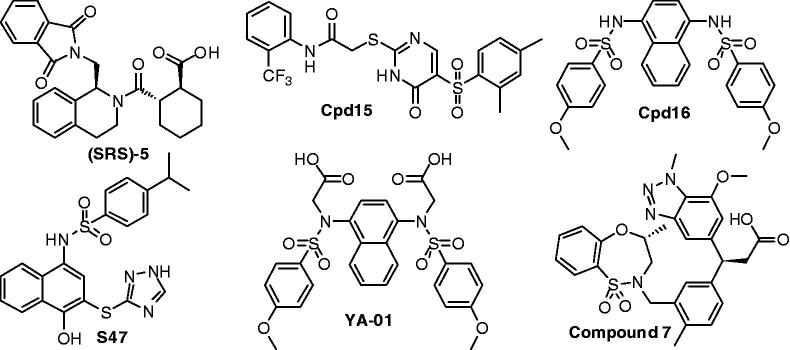
Recently reported small-molecule Keap1–Nrf2 PPI inhibitors.

**Figure 2.**
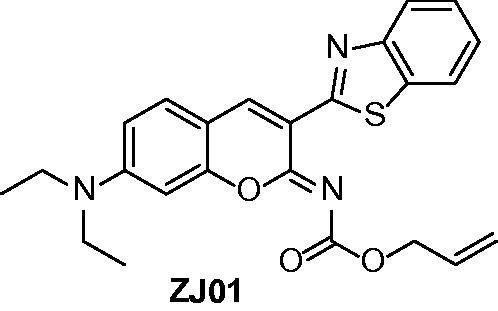
Structure of **ZJ01**.

Recently, small molecules (Figure 1) directly targeting the Keap1–Nrf2 PPI have been reported by several research groups^18–22^. However, the structural diversity of current Keap1–Nrf2 PPI inhibitors is still limited. In our project to discover Keap1–Nrf2 PPI inhibitors, the novel small molecule **ZJ01** with an iminocoumarin-benzothiazole scaffold (Figure 2) was recently screened out from our in-house compound library based on fluorescence polarization (FP) assay. In the present study, we describe the identification of **ZJ01** as a new Keap1–Nrf2 PPI inhibitor and further investigate its *in vitro* and *in vivo* cytoprotective effects on LPS-induced cardiomyopathy.

## Methods

### Chemistry

#### General experimental procedures

Commercially available reagents were used without further purification. Organic solvents were evaporated with reduced pressure using a Buchi rotary evaporator. Reactions were monitored by TCL using Yantai Jiangyou (China) GF254 silica gel plates. Silica gel column chromatography was performed on silica gel (300–400 mesh) from Qingdao Haiyang (China). The NMR spectra were measured on Bruker Avance 600 spectrometer. Chemical shifts were expressed in δ (ppm) and coupling constants (*J*) in Hz using solvent signals as internal standards (CDCl_3_, δ_H_ 7.26 ppm; δ_C_ 77.0 ppm). ESI-MS was recorded on an Agilent 6460 Triple Quard LC/MS, and HRMS spectrum were recorded on an Agilent Q-ToF 6520.

#### 3-(Benzo[d]thiazol-2-yl)-N,N-diethyl-2-imino-2H-chromen-7-amine (3)

To a solution of (1,3-benzothiazol-2-yl)acetonitrile (**1**) (165 mg, 0.86 mmol) and 4-4-(diethylamino)-2-hydroxybenzaldehyde (**2**) (150 mg, 0.86 mmol) in MeOH (10 ml) was added a drop of piperazine. The mixture was stirred at 20 °C for 16 h under N_2_, the precipitate was collected by filtration, washed with methanol, and dried under high vacuum to afford **3** ^23^ (255 mg, 85%). This compound was used in the next step without further purification. ^1^H NMR (600 MHz, CDCl_3_) δ 8.25 (1H, br), 8.01 (1H, d, *J* = 8.1 Hz), 7.90 (1H, d, *J* = 7.9 Hz), 7.47 (1H, dd, *J* = 8.1, 7.1 Hz), 7.35 (1H, dd, *J* = 7.9, 7.1 Hz), 7.29 (1H, d, *J* = 8.7 Hz), 6.48 (1H, dd, *J* = 8.7, 2.4 Hz), 6.40 (1H, brs), 3.42 (4H, q, *J* = 7.1 Hz), 1.22 (6H, t, *J* = 7.1 Hz). ESIMS *m/z*: 350.1 [M + H]^+^.

#### (Z)-allyl (3-(benzo[d]thiazol-2-yl)-7-(diethylamino)-2H-chromen-2-ylidene)carbamate (ZJ01)

To a solution of **3** (60 mg, 0.17 mmol) in DCM (5 ml) was added Et_3_N (24 μL, 0.17 mmol). The solution was cooled to 0 °C for 1 h, and then allyl carbonochloridate (10 μL, 0.17 mmol) was added. The mixture was stirred overnight, concentrated, and purified by silica gel column (petroleum ether–ethyl acetate, 4:1) to afford pure **ZJ01** as a red powder (18 mg, 25%). mp 148–150 °C. ^1^H NMR (600 MHz, CDCl_3_) δ 8.82 (1H, s), 8.02 (1H, brd, *J* = 8.1 Hz), 7.92 (1H, brd, *J* = 7.9 Hz), 7.49 (1H, ddd, *J* = 8.1, 7.2, 1.2 Hz), 7.42 (1H, d, *J* = 8.8 Hz), 7.35 (1H, ddd, *J* = 7.9, 7.2, 1.0 Hz), 6.63 (1H, dd, *J* = 8.8, 2.3 Hz), 6.47 (1H, d, *J* = 2.3 Hz), 6.10 (1H, ddt, *J* = 17.2, 10.5, 5.6 Hz), 5.48 (1H, ddt, 17.2, 1.8, 1.4 Hz), 5.31 (1H, ddt, *J* = 10.5, 1.8, 1.4 Hz), 4.86 (2H, ddd, *J* = 5.6, 1.4, 1.4 Hz), 3.45 (4H, q, *J* = 7.1 Hz), 1.24 (6H, t, *J* = 7.1 Hz). ^13^C NMR (125 MHz, CDCl_3_) δ 161.5, 159.6, 155.8, 153.1, 152.4, 151.9, 139.8, 137.0, 132.7, 130.8, 126.1, 124.5, 122.3, 121.6, 118.2, 114.2, 109.9, 108.6, 97.0, 67.0, 45.1, 12.7. ESIMS *m/z*: 434.1 [M + H]^+^. HR-ESIMS: [M + H]^+^ calcd for C_24_H_24_N_3_O_3_S^+^ 434.1533, found 434.1546.

#### (Z)-N-(3-(benzo[d]thiazol-2-yl)-7-(diethylamino)-2H-chromen-2-ylidene)acrylamide (ZJ02)

To a solution of **3** (60 mg, 0.17 mmol) in DCM (5 ml) was added Et_3_N (24 μL, 0.17 mmol). The solution was cooled to 0 °C for 1 h, and then acryloyl chloride (14 μL, 0.17 mmol) was added. The mixture was stirred overnight, concentrated, and purified by silica gel column (petroleum ether–ethyl acetate, 4:1) to afford pure **ZJ01** as a red powder (27 mg, 39%). mp 188–190 °C. ^1^H NMR (600 MHz, CDCl_3_) δ 8.80 (1H, s), 8.02 (1H, d, *J* = 8.1 Hz), 7.91 (1H, d, *J* = 7.9 Hz), 7.47 (1H, dd, *J* = 7.4, 7.9 Hz), 7.39 (1H, d, *J* = 8.8 Hz), 7.35 (1H, dd, *J* = 7.4, 8.1 Hz), 6.59 (1H, dd, *J* = 8.8, 2.3 Hz), 6.54–6.46 (2H, m), 6.44 (1H, d, *J* = 2.0 Hz), 5.97 (1H, dd, *J* = 2.3, 9.3 Hz), 3.41 (4H, q, *J* = 7.2 Hz), 1.24 (6H, t, *J* = 7.2 Hz). ^13 ^C NMR (125 MHz, CDCl_3_) δ 176.7, 161.7, 155.9, 152.4, 151.9, 150.8, 139.6, 136.9, 134.6, 130.8, 130.3, 126.1, 124.5, 122.3, 121.5, 114.2, 109.8, 108.6, 97.0, 45.1, 12.7. ESIMS *m/z*: 404.1 [M + H]^+^. HR-ESIMS: [M + H]^+^ calcd for C_23_H_22_N_3_O_2_S^+^ 404.1427, found 404.1429.

### Biology

#### FP assay

Determination of equilibrium dissociation constants *K*_D2_ for the tested compound was performed using the FP assay by fitting the displacement curves as described in our previous study^20^. The FP was measured on Biotek Syngergy 2 microplate reader with 485 nm excitation and 535 nm emission after a 60 min incubation at room temperature. The *K*_D1_ value is determined with FITC − βAla − DEETGEF − OH as the fluorescence probe.

#### Surface plasmon resonance (SPR) analysis

Surface plasmon resonance analysis was performed on a Biacore TM T200 instrument (GE Healthcare, Salem, CT, USA). Keap1 proteins were immobilized on a CM5 chip via EDC/NHS-mediated crosslinking reaction. Tested compound was diluted in PBS at concentrations ranging from 0.25 to 64 µM. The analysis was then performed according to the protocol provided by GE Healthcare. In each analysis, the middle concentration was duplicated at the end of the wash run to confirm the stability of the sensor surface. The parameters of SPR were set as follow: flow rate, 30 µL/min; contact time, 120 s; disassociation time, 120 s. Affinity curve fitting was performed with the Biacore T200 Evaluation Software using a steady-state affinity model (1:1) to calculate the disassociation constant (*K*_d_). Detailed SPR measurement data are shown in Table 1.

**Table 1.** SPR measurement data of Keap1 with **ZJ01.**

Protein	Ligand	*K*_d_ (µM)	*R*_max_ (RU)	Offset (RU)	Chi^2^ (RU^2^)
Keap1	**ZJ01**	48.1	58.51	0.2069	0.236

#### Cell culture and treatment

H9c2 cells, a rat ventricular myoblast cell line, were purchased from the Cell Bank of Chinese Academy of Sciences (Shanghai, China). The Cells were cultured in Dulbecco’s modified Eagle’s medium with 10% bovine calf serum and then were maintained at 37 °C under humidified conditions with 5% CO_2_. Cells were seeded in appropriate dishes (30,000 cells/ml). After culture for 24 h, the cells were treated with different concentration of inhibitors and incubated with or without 1 μg/ml of LPS for indicated time.

#### Mice and treatments

Male C57BL/6 mice (eight weeks old) were purchased from the Experimental Animal Center of Shandong University (Jinan, Shandong, China). All *in vivo* experiments followed the ARRIVE guidelines^24^. Inhibitors or LPS was dissolved in DMSO: normal saline (1:100). The control group was injected intraperitoneally with equal DMSO and normal saline. C57BL/6 mice were challenged with different concentrations of ZJ01 or S47 overnight for approximately 12 h after being treated intraperitoneally with or without 4 mg/kg of LPS. At the end of treatment, all mice were euthanized by intravenous lateral tail vein injection of keta-mine/xylazine (Sigma-Aldrich, Saint Louis, MO, USA, 150 mg/kg ketamine combined with 10 mg/kg xylazine). The left ventricles were collected for western blotting or real-time PCR assay.

#### Western blotting assay

Protein Extraction Kit (Beyotime, China) was used to isolate the nuclear and cytosol protein of H9c2 cells and left ventricular cells of C57BL/6 mice according to the protocol. Then the collected protein was stored at −80 °C until use. Equal amounts of protein were applied to 12% SDS-polyacrylamide gel. Proteins in gels were electroblotted onto poly-vinylidene difluoride membranes. After blocking at room temperature for 1 h, the membranes were probed with primary antibodies overnight at 4 °C. After three washes in TBST, membranes were incubated with peroxidase-conjugated secondary antibodies for 1 h at room temperature, and proteins were detected by use of an enhanced chemiluminesence detection kit.

#### Immunofluorescence analysis

Treated cells were fixed in 4% paraformaldehyde (w/v) for 30 min at room temperature, then incubated with normal goat serum (1:30) for 20 min and Nrf2 antibodies (1:100) overnight at 4 °C. Cells were washed with PBS for three times, then incubated with corresponding secondary antibodies (1:200) for 1 h at 37 °C. Fluorescence was detected by laser scanning confocal microscopy (Leica, Wetzlar, Germany).

#### DCFH-DA staining for analysis of intracellular ROS activity level

H9c2 Cells (1 × 10^4^ per well) were seeded in black bottomed 96-well culture plate and cultured for 24 h in a CO_2_ incubator at 37 °C. After treatment, cells were incubated with 10 mM DCFH-DA for 30 min at 37 °C. After washing with PBS for three times, fluorescence intensity was measured with a multi-well microplate reader at an emission wavelength of 528 nm and at an excitation wavelength of 485 nm. All the values were expressed as percentage fluorescence intensity relative to the control.

#### Real-time PCR

Total RNAs were extracted from treated cells or left ventricle of C57BL/6 mice with TriZol Reagent (Invitrogen Life Technologies, Waltham, MA, USA). RNA (250–500 ng) was reverse-transcribed using the Prime Script RT reagent kit with gDNA Eraser (DRR047, TAKARA) according to the manufacturer’s instructions. The RT-PCR reactions were performed using QuantiTect SYBR Green PCR kit (QIAgen, Dusseldorf, Germany) and LightCycler 2.0 system (Roche Diagnostics, Shanghai, China). Reactions were carried out in a 25 μl volume containing 12.5 μl of 2 × SYBR Green PCMaster Mix. The fold-changes for RNA level were calculated using the MxPro software (Version 4.00, Stratagene, San Diego, CA, USA).

#### Molecular docking simulation

To obtain the starting structure of Keap1/**ZJ01** for simulation, molecular docking was performed with Autodock-4^25^. For the docking calculations we obtained the initial Keap1 complex crystal structures from the Protein Data Bank (www.pdb.org)-(PDB id: 4IQK). Prior to docking, all the water molecules have been removed from Keap1 as none of them plays any role in inhibitor binding. AutoDockTools have been used to prepare the enzyme prior to the docking. Gasteiger^26^ partial charges have been assigned to both the inhibitor and enzyme atoms. The docking sampled the ligands in a 126 × 126 × 126 grid with 0.375 Å resolution that was positioned to encompass the Keap1 binding gorge. The quality of the re-docking has been quantified by the root meansquare deviation (RMSD) between the top-scoring docked pose from each docking method and the corresponding X-ray crystal structure. Prior to docking all X-ray ligand structures were compared with PubChem (www.pubchem.com) structures for any missing atoms and then optimized using the program LigPrep from the Schrödinger (Schrödinger Release 2015–2: LigPrep, Schrödinger, LLC, New York, NY, 2015). The optimization is based on molecular mechanics and used the MMFFS force field^27^. The docking results were analysed with the programs AutoDockTools^25^, DOCKRES^28^ and VMD^29^. Molecular graphics figures were prepared with the LigPlot + program^30^.

#### Molecular dynamics simulations

Molecular dynamics simulations were performed on the Keap1/**ZJ01** complex obtained from molecular docking. Before simulations, the protonation states of ionisable residues were determined using the H++ program^31^. The complex model was surrounded by a periodic box of transferable intermolecular potential 3P^32^ water molecules that extend 12 Å from the protein atoms. Counter-ions were added to neutralize the simulation system. Molecular dynamics simulations were performed using the GROMACS 5.0.4 package^33^ with isothermal-isobaric (NPT) ensemble and periodic boundary condition. The CHARMM36-CAMP force field^34^ was used for the protein, ions and water molecules. Atom charges of **ZJ01** were calculated using the restrained electrostatic potential method^35^ encoded in the AMBER suite of programs^36^ at the RHF/6-31G* level. Covalent and non-bonded parameters for **ZJ01** atoms were assigned by analogy or interpolation from those already present in the AMBER force field.

Energy minimizations were first performed to relieve unfavourable contacts in the system, followed by equilibration steps of 1 ns in total to equilibrate the complex. Subsequently, 50 ns production runs were performed. The temperature of the system was maintained at 300 K using the v-rescale method^37^ with a coupling time of 0.1 ps. The pressure was kept at 1 bar using the Parrinello–Rahman^38^ with *τ*_p_ = 1.0 ps and a compressibility of 4.5 × 10^−5 ^bar^−1^. SETTLE^39^ constraints and LINCS^40^ constraints were applied to the hydrogen-involved covalent bonds in water molecules and in other molecules, respectively, and the time step was set to 2 fs. Electrostatic interactions were calculated with the particle-mesh Ewald (PME) algorithm^41^ with a real-space cut-off of 1.2 nm.

#### Binding free energy calculation by MMPBSA method

Based on the equilibrated dynamic trajectory, the binding free energy of the complex was calculated using the MM-PBSA method encoded in the AMBER 14.0 program. A total of 2500 snapshots from the trajectory were extracted every 20 ps, and the MM-PBSA calculation was performed on each snapshot using the MMPBSA.py module in AMBER 14.0.

## Results

### Chemistry

**Scheme 1.**
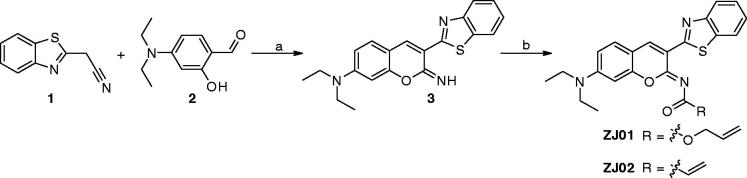
Reagents and conditions: (a) a drop of piperazine, MeOH, r.t., 16 h; (b) DCM, Et_3_N, allyl carbonochloridate, 0 °C, overnight for **ZJ01**; DCM, Et_3_N, acryloyl chloride, 0 °C, overnight for **ZJ02**.

The synthetic route for **ZJ01** is outlined in Scheme 1. Briefly, condensation of (1,3-benzothiazol-2-yl)acetonitrile (**1**) with 4-(diethylamino)-2-hydroxybenzaldehyde (**2**) readily produced the intermediate **3** ^23^, which was then converted to the target compounds by reaction with allyl carbonochloridate or acryloyl chloride.

### Biological evaluation

#### Hits identification and confirmation

**Figure 3.**
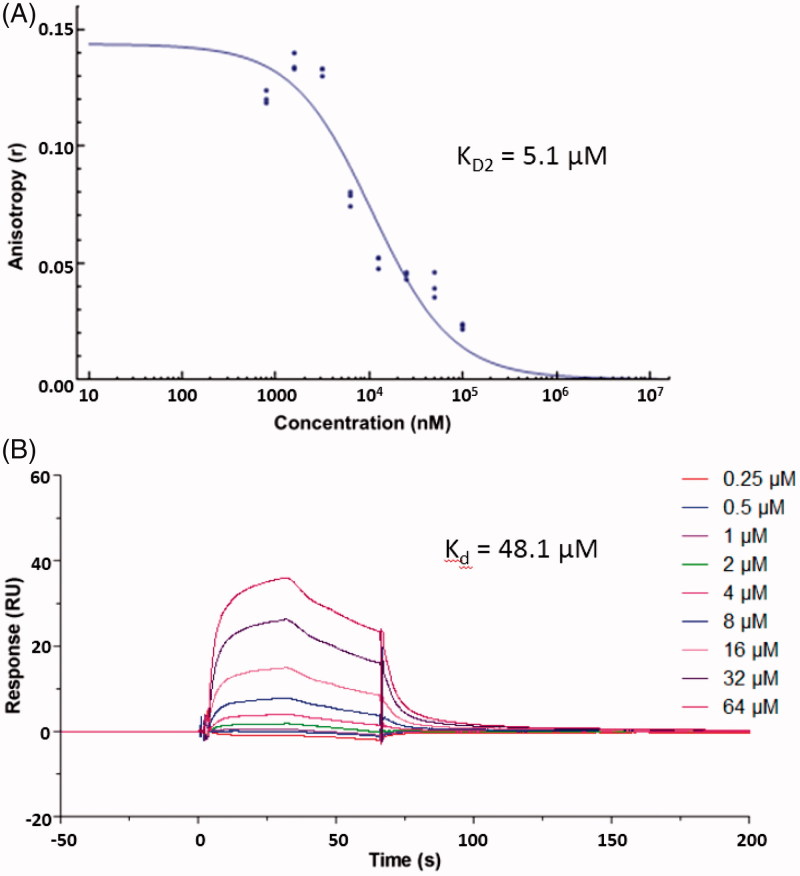
(A) Dose–response curve of **ZJ01** in FP assay. (B) SPR sensorgrams of **ZJ01**.

To identify Keap1–Nrf2 PPI inhibitors, our in-house library of 569 compounds was screened for their activity (*K*_D2_, *K*_D1_ is the equilibrium dissociation constant of the fluorescent probe) in FP assay^20^ at the concentration of 100 μM, with the previously identified Keap1–Nrf2 PPI inhibitor **S47** ^20^ as a positive control. The assay results revealed synthetic compound **ZJ01** to have the best inhibitory activity, demonstrating 81.25% inhibition of Keap1-Nrf2 PPI interaction at 100 μM. However, an analogue (**ZJ02**, Scheme 1) with the same core structure displayed only 20% inhibition in the initial testing, indicating that the side chain play a vital role in their activity. Compound **ZJ01** was then retested to determine its *K*_D2_, which was found to be 5.1 μM (Figure 3(A)), slightly better than that of **S47** (*K*_D2_ ≈ 7.6 μM, close to the reported 2.9 μM)^20^. Later, a surface plasmon resonance (SPR) competition assay^42^ was employed to confirm the interaction between **ZJ01** and the Keap1 Kelch domain. As shown in Figure 3(B), **ZJ01** displayed an extracellular binding affinity with Keap1 with an equilibrium dissociation constant (*K*_d_) of 48.1 μM, somewhat weaker than that of **S47** (*K*_d_ ≈ 39.4 μM). The equilibrium binding curve fits are shown in Figures S14 and S15 (ESI†). These results indicated that **ZJ01** can directly bind to Keap1 and disrupt the Keap1–Nrf2 interaction.

#### Molecular modelling study

**Figure 4.**
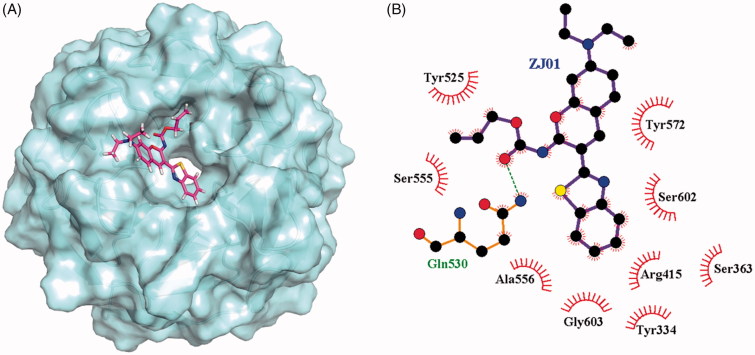
(A) Putative binding mode of **ZJ01** to Keap1. (B) Schematic diagram showing interactions between **ZJ01** and Keap1. Residues involved in hydrogen bonds and hydrophobic interactions are shown as sticks and starbursts, respectively. Molecular graphics figures were prepared with the LigPlot + program.

To investigate the possible binding mode of **ZJ01** to the Keap1 Kelch domain, molecular docking was conducted using Autodock-4^25^. The binding mode of **ZJ01** to Keap1 is shown in Figure 4. A 50 ns molecular dynamics (MD) study, discussed in the following section, did not indicate any instability of the docked Keap1/**ZJ01** complex. Similar docking was also performed for Keap1/**ZJ02**. Compared with **ZJ01**, the result (Figure S16, ESI†) indicated that **ZJ02** had a totally different spatial orientation at the Keap1–Nrf2 interaction surface, which possibly resulted from the protonated nitrogen atom of amide in **ZJ02**, which remained unprotonated in **ZJ01**. This was speculated to be the reason that these two compounds exhibited different activities in the FP assay.

### MD simulation

#### Steady RMSDs values indicate that the MD trajectory is reliable

**Figure 5.**
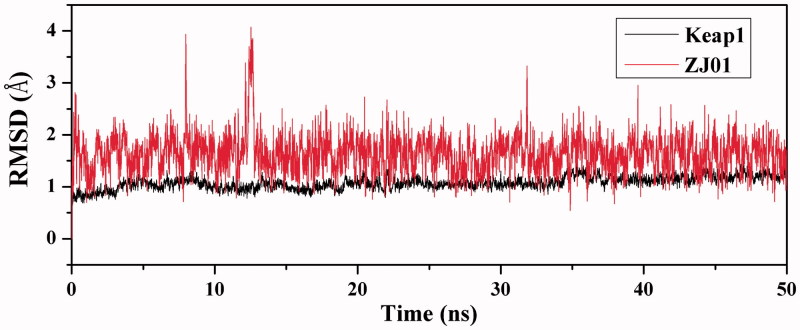
Time dependencies of RMSDs for the backbone atoms of the Keap1–**ZJ01** complex.

To explore the dynamic traits of the Keap1/**ZJ01** complex, a 50 ns MD simulation was performed on the complex model obtained by molecular docking. To examine the structural stability of the complex during MD simulations, the time evolution of weighted root-mean-square deviations (RMSDs) for backbone atoms of the Keap1 protein and heavy atoms of **ZJ01** from their initial positions (*t* = 0) was calculated. As illustrated in Figure 5, RMSD values of the protein backbone were found to be between 0.5 and 1.2 Å during simulations. Steady RMSD values for the heavy atoms of the protein and **ZJ01** indicated well-equilibrated states of the system. The steady RMSD values showed the reliability of the MD trajectories and suggested the suitability for post analysis.

#### H-bond and hydrophobic interaction analyses revealed a well-defined substrate pocket

To probe the molecular interactions between Keap1 and **ZJ01**, the H-bond and hydrophobic interactions of the complex were analysed based on MD trajectories. As shown in Table 2, there is only one conserved H-bond between Keap1 and **ZJ01** in the complex. Additionally, the non-polar contacts between active site residues and **ZJ01** cannot be ignored due to the fact that Keap1 has a relatively hydrophobic pocket. Thus, the occupancy rates of hydrophobic interactions between active site residues and **ZJ01** were calculated based on MD trajectories. The result demonstrated that residues Tyr334, Arg415, Tyr525, Ala556, Tyr572, and Ser602 have more intense hydrophobic interactions with **ZJ01** (Table 3). Taken together, these results indicated extensive polar and non-polar interactions between Keap1 and **ZJ01**.

**Table 2.** Hydrogen bond existing in the complex and its occupancy during MD simulation.

H-bond donor	H-bond acceptor	Occupancy rate (%)^a^
Gln530:NE2	ZJ01:O2	99

a Only H-bond occupancies >50% are shown.

**Table 3.** Residues involved in hydrophobic interactions with **ZJ01** during MD simulation and their corresponding occupancies rates.

Residue	Occupancy rate (%)^a^
Tyr 334	94
Arg 415	81
Tyr 525	96
Ala 556	89
Tyr 572	99
Ser 602	51

a Only hydrophobic interaction occupancies >50% are shown.

### Binding free energy analysis

To further explore the molecular interactions between Keap1 and **ZJ01**, the binding free energy values of the Keap1/**ZJ01** complex were calculated using the MM-PBSA method encoded in the AMBER 14 program (in Table 4)^43^. The binding free energy (Δ*G*) value of –26.10 kcal/mol suggested a stable binding between **ZJ01** and Keap1.

**Table 4.** Binding free energy values of the Keap1/**ZJ01** complex.

Inhibitor	Δ*G*_gas_^a^	Δ*G*_solv_^b^	Δ*G*^c^
**ZJ01**	−60.71 ± 4.77	34.62 ± 3.89	−26.10 ± 2.72

All calculated values are given in kcal/mol.

a Δ*G*_gas_ represents the binding free energy in vacuum.

b Δ*G*_solv_ represents the solvation free energy change calculated by the MM-PBSA method.

c Δ*G* = Δ*G*_gas_ + Δ*G*_solv_.

#### Nrf2–ARE pathway activation in H9c2 cardiac cells

**Figure 6.**
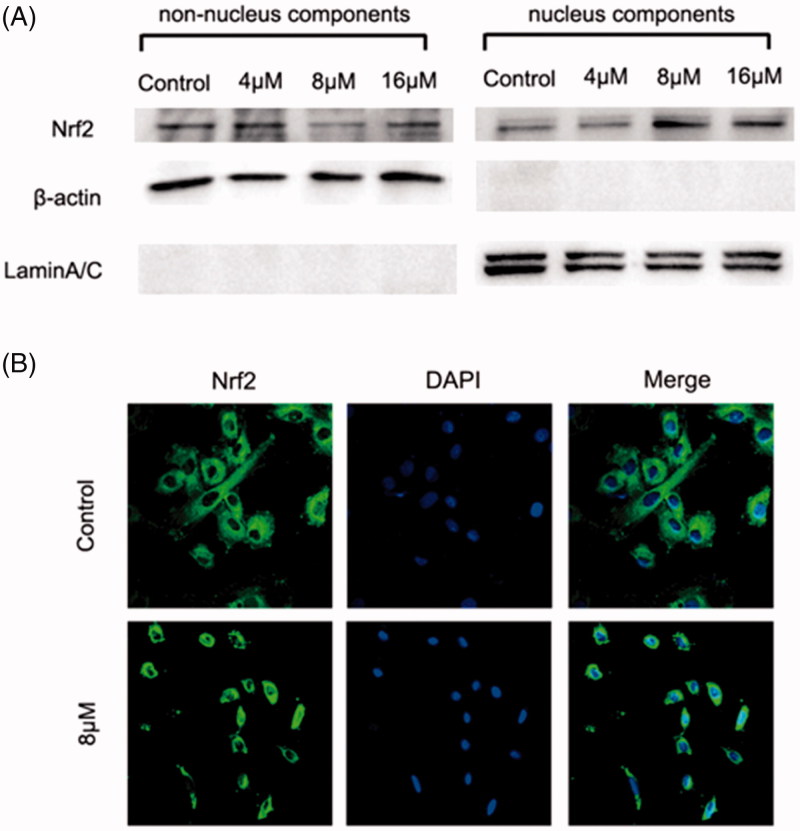
**ZJ01** induced nuclear translocation of Nrf2 in H9c2 cells. H9c2 cells were treated with different concentrations of **ZJ01** for 6 h. (A) Western blotting analysis of nuclear and non-nuclear Nrf2 protein levels. (B) Immunofluoresence staining analysis of Nrf2 localization. Nuclei were counterstained with PI.

The above results demonstrated that **ZJ01** was an effective direct Keap1–Nrf2 PPI inhibitor. Previous studies have confirmed that Keap1–Nrf2 PPI inhibitors can make Nrf2 detach from Keap1 and subsequently translocate to the nucleus to activate the anti-oxidant defense system^20^ ^,^ ^21^. Therefore, the effect of **ZJ01** on the distribution of Nrf2 in H9c2 cardiac cells was investigated. Western blotting analysis indicated that **ZJ01** increased the nuclear Nrf2 protein levels and decreased the non-nuclear Nrf2 protein levels at 8 μM (Figure 6(A)). Meanwhile, the immunofluorescence assay demonstrated that Nrf2 translocated into the nucleus after the cells were treated with **ZJ01** (Figure 6(B)) or **S47** (Figure S17).

**Figure 7.**
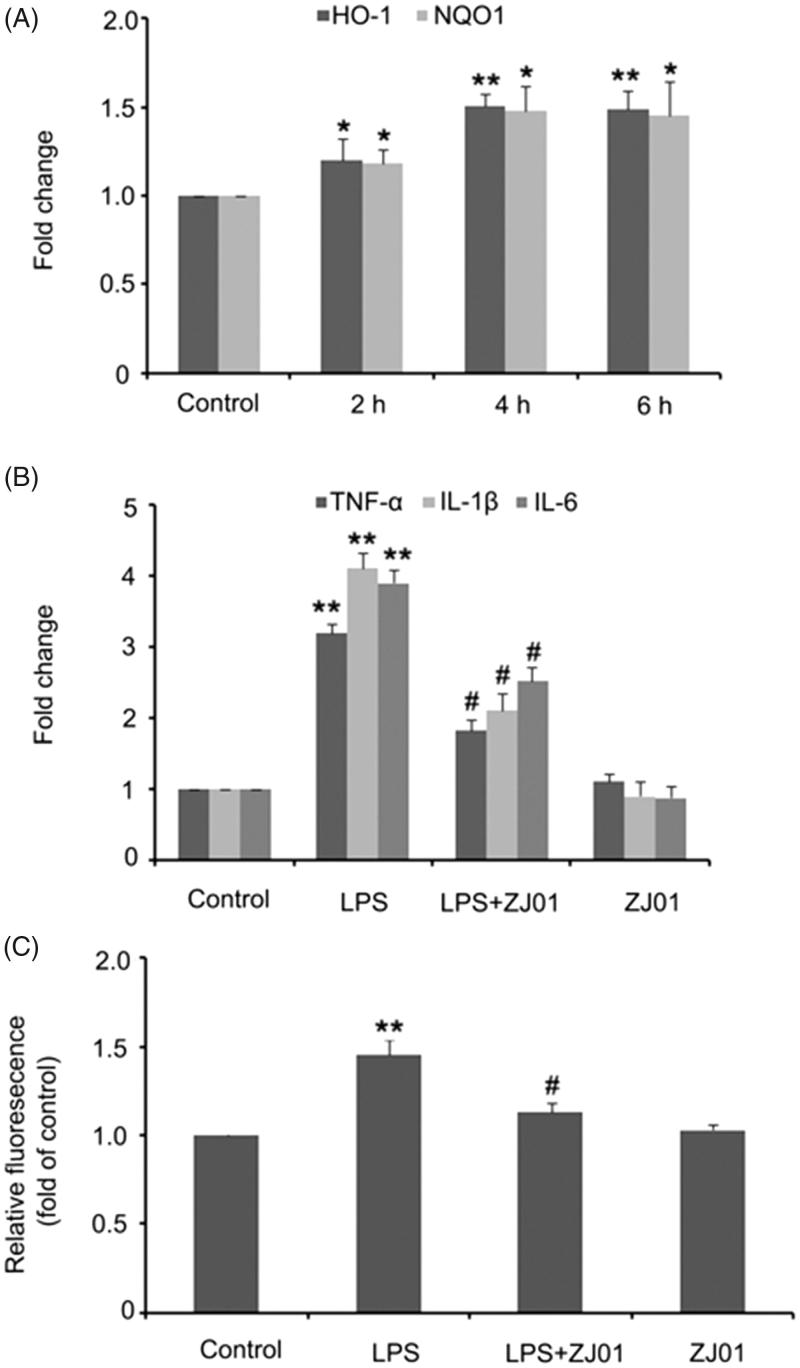
**ZJ01** increased the expression of anti-oxidant genes and alleviated LPS-induced production of pro-inflammatory cytokines and ROS in H9c2 cells. (A) RT-PCR analysis of anti-oxidant genes HO-1 and HQO1 after treatment of H9c2 cells with 8 μM **ZJ01** for 6h. (B,C) H9c2 cells were stimulated with 1 μg/ml LPS and treated with or without 8 μM **ZJ01** for 6 h. (B) The expression levels of pro-inflammatory cytokines TNF-α, IL-1β and IL-6 were determined by RT-PCR. (C) The intracellular ROS levels were examined by DCHF. *p < .05, **p < .01 vs. control, #*p* < .05 vs. LPS group. *n* = 3.

The nuclear Nrf2 could bind to the ARE of target genes, which then resulted in transcriptional induction and chemoprotection effects. Thus, the mRNA levels of Nrf2 target anti-oxidant genes, such as HO-1 and NQO1, were further examined in H9C2 cells after exposure to **ZJ01**. It was found that **ZJ01** could significantly increase the HO-1 and NQO1 mRNA levels (Figure 7(A)). Altogether, these results revealed that **ZJ01** activated the Nrf2–ARE pathway in H9c2 cardiac cells and might be a protective agent in H9c2 cardiac cells.

##### Anti-inflammatory response and anti-oxidative stress ability assay in H9c2 cardiac cells

Oxidative stress and inflammatory responses are responsible for the deterioration of sepsis-induced cardiomyopathy. LPS has been used to induce an inflammatory response and oxidative stress in H9c2 cells, which are typically used for *in vitro* investigations^4^ ^,^ ^44^. Inflammatory cytokines, including tumour necrosis factor (TNF)-α, interleukin (IL)-1α, IL-1β, and IL-6, promote the development of sepsis-induced cardiomyopathy^45^. Previously published studies indicated that LPS could increase the production of IL-1β, IL-6, and TNF-α in H9c2 cardiomyocytes and primary cardiomyocytes^4^ ^,^ ^46^, and additional evidence postulated that LPS-induced ROS could lead to the activation of intracellular signalling pathways and transcription factors, and subsequently induce the production of inflammatory mediators, including TNF-α, IL-1β and IL-6^7^ ^,^ ^47–49^. Therefore, inhibiting oxidative stress and blocking inflammatory signalling may produce beneficial effects in the dysfunctional heart. The effects of **ZJ01** on the production of TNF-α, IL-1β, IL-6 and ROS were thus examined in LPS-stimulated H9c2 cells as well as the *in vitro* model.

Consistent with previously published data from other groups, our results showed that 1 μg/ml LPS induced a significant increase in mRNA levels of TNF-α, IL-1β and IL-6 in H9c2 cardiac cells. The 8 μM volume of **ZJ01** or **S47**, which could effectively activate Nrf2, was applied to test its inhibitory effect on the production of TNF-α, IL-1β and IL-6 expression after exposure to LPS. It was observed that **ZJ01** and **S47** markedly alleviated LPS-induced production of these pro-inflammatory cytokines at the mRNA level (Figure 7(B); Figure S18A). Intracellular ROS levels were measured by dichloro-dihydro-fluorescein diacetate (DCFH-DA). The results showed that the ROS levels were significantly upregulated after treatment with 1 μg/ml LPS, and this increase could be blocked by 8 μM **ZJ01** (Figure 7(C)). However, 8 μM **S47** could not inhibit the elevated ROS levels induced by LPS (Figure S18B). The above results revealed that **ZJ01** and **S47** had the same anti-inflammatory effect, while **ZJ01** had better anti-oxidant effects on LPS-treated myocardial cells. As ZJ01 is an effective Keap1–Nrf2 PPI inhibitor, we speculated ZJ01 might inhibit LPS-induced cardiomyopathy through disrupting Keap1–Nrf2 PPI which could induce Nrf2 nuclear accumulation and thus blocking oxidative and inflammatory signalling.

##### In vivo studies

**Figure 8.**
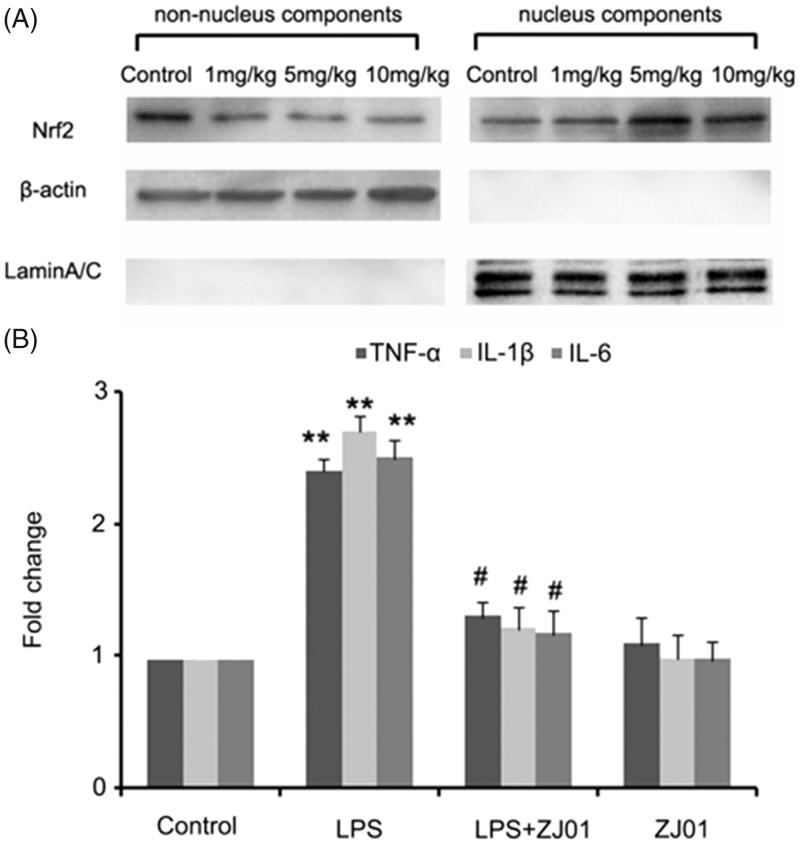
**ZJ01** induced the Nrf2 nuclear accumulation and inhibited LPS-induced inflammatory cytokines production of left ventricular cells *in vivo*. (A) C57BL/6 mice were treated intraperitoneally with different concentrations of **ZJ01** for 12 h. The nuclear and non-nuclear Nrf2 protein levels of left ventricular cells were determined by Western blotting technology. (B) C57BL/6 mice were treated intraperitoneally with 4 mg/kg of LPS and then with 5 mg/kg **ZJ01** for 12 h. RT-PCR analysis of the expression of pro-inflammatory cytokines IL-1β, IL-6, and TNF-α. **p < .01 vs. control, #*p* < .05 vs. LPS group. *n* = 3.

As left ventricular inflammation is an important feature of cardiac injury induced by LPS^16^, to examine whether **ZJ01** and **S47** were able to confer a cardiac protective effect *in vivo*, C57BL/6 mice were challenged with different concentrations of **ZJ01** or **S47** overnight for approximately 12 h after being treated intraperitoneally with or without 4 mg/kg of LPS, a dose commonly used for experimental models of septic cardiomyopathy in animals^16^. Then, the left ventricles were collected for further studies. Consistent with the *in vitro* experimental results, western blotting assay demonstrated that ZJ01 increased the nuclear Nrf2 protein levels and decreased the non-nuclear Nrf2 protein levels at 5 mg/kg or 10 mg/kg concentration suggesting ZJ01 could induce Nrf2 nuclear accumulation in left ventricular cells (Figure 8(A)). Real-time PCR results showed that inhibit LPS-induced inflammatory cytokines including TNF-α, IL-1β and IL-6 at the mRNA level in in left ventricular cells *in vivo* (Figure 8(B)). Compared to **ZJ01**, **S47** could induce the Nrf2 nuclear accumulation at a 10 mg/kg concentration (Figure S19), but did not exhibit obvious effects on Nrf2 nuclear accumulation at the lower 5 mg/kg concentration. This result suggested that **ZJ01** might be more effective than **S47** in activating Nrf2 of cardiomyocytes *in vivo*.

## Conclusions

In this study, a new Keap1–Nrf2 PPI inhibitor **ZJ01** with an iminocoumarin-benzothiazole core was identified via FP and SPR assays. The amide side chain of **ZJ01** was found to have an important role in its activity. **ZJ01** could inhibit LPS-induced production of pro-inflammatory cytokines (TNF-α, IL-1β and IL-6) and ROS through activating the Nrf2–ARE pathway in H9c2 cells. *In vivo*, **ZJ01** also induced Nrf2 nuclear accumulation and inhibited LPS-induced inflammatory cytokine production in cardiomyocytes. The biological data, together with computational results, indicated that **ZJ01** could serve as a new template for generating direct Keap1–Nrf2 PPI inhibitors and possessed great potential in septic cardiomyopathy therapy.

## Supplementary Material

IENZ_1461856_Supplementary_Material.pdf
