# Supplementary material for: Identification of a novel small-molecule Keap1–Nrf2 PPI inhibitor with cytoprotective effects on LPS-induced cardiomyopathy
Source: J Enzyme Inhib Med Chem. 2018 Apr 25;33(1):833–41. doi: 10.1080/14756366.2018.1461856 (PMC6009974; doi:10.1080/14756366.2018.1461856)
Supplement: IENZ_1461856_Supplementary_Material.pdf [file IENZ_A_1461856_SM1140.pdf]

Identification of a novel small-molecule Keap1-Nrf2 PPI inhibitor with cytoprotective effects on LPS-induced cardiomyopathy

Cheng-Shi Jiang‡[a], Chun-Lin Zhuang‡[b], Kongkai Zhu‡[a], Juan Zhang[cd], Luis Alexandre Muehlmann[cd], João Paulo Figueiró Longo[c], Ricardo Bentes Azevedo[c], Wen Zhang[b], Ning Meng[a]* and Hua Zhang[a]*

[a] School of Biological Science and Technology, University of Jinan, Jinan 250022, China

[b] School of Pharmacy, Second Military Medical University, Shanghai 200433, China

[c] Faculty of Ceilandia, University of Brasilia, Brasilia 72220275, Brazil

[d] Institute of Institute of Biological Sciences, University of Brasília, Brasilia 70910900, Brazil

Chemistry

General: commercially available reagents were used without further purification. Organic solvents were evaporated with reduced pressure using a Buchi R-100 rotary evaporator. Reactions were monitored by TLC using Yantai Jiangyou (China) GF254 silica gel plates. Silica gel column chromatography was performed on silica gel (300-400 mesh) from Qingdao Haiyang Ltd. (China). NMR spectra were measured on Bruker Avance 600 spectrometer. Chemical shifts were expressed in δ (ppm) and coupling constants (*J*) in Hz using solvent signals as internal standards ($CDCl_3$, δ_H 7.26 ppm; δ_C 77.0 ppm). ESI-MS was recorded on an Agilent 6460 Triple Quard LC/MS, and HR-ESI-MS spectrum were recorded on an Agilent Q-TOF 6520.

Purities of synthetic compounds were analyzed on an Agilent 1260 HPLC using a ZDRBAX SB-C18 column (4.6×150 mm), with UV detection at 254 nm and a linear A-B gradient (A: MeOH; B: H_2O) at a flow rate of 1 mL/min.

Figure S1 ^{1}H NMR spectrum of 3

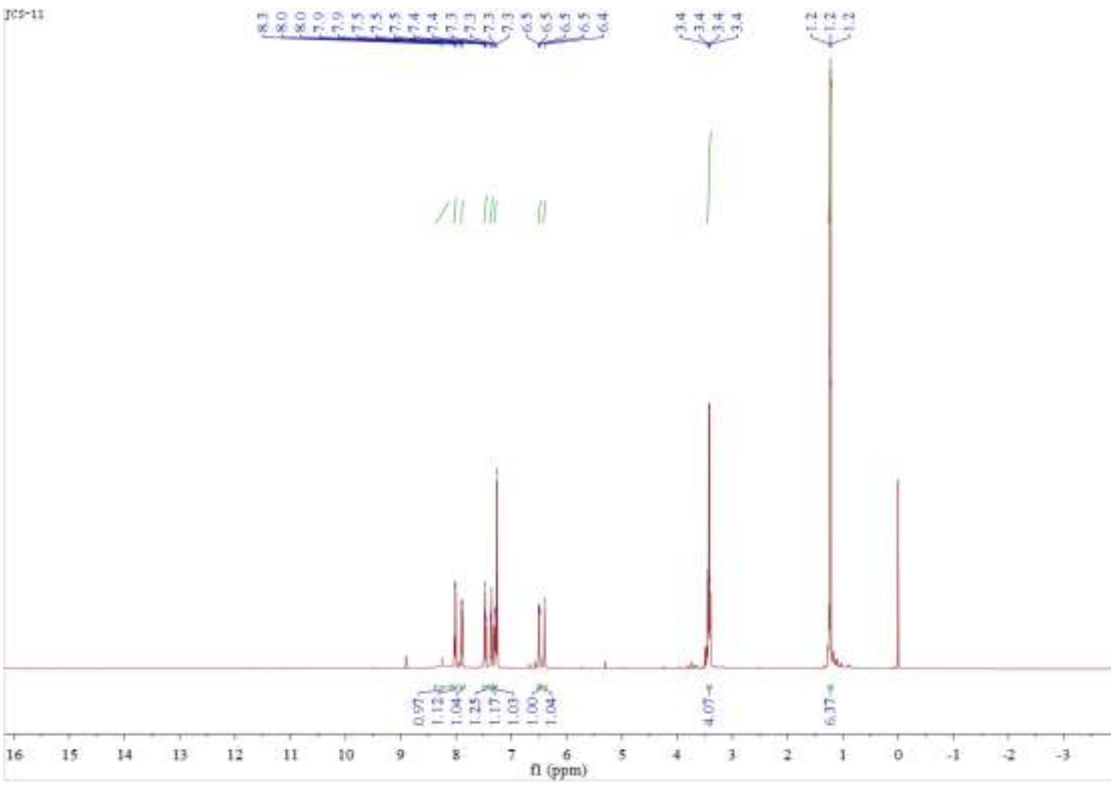

Figure S2 MS spectrum of 3

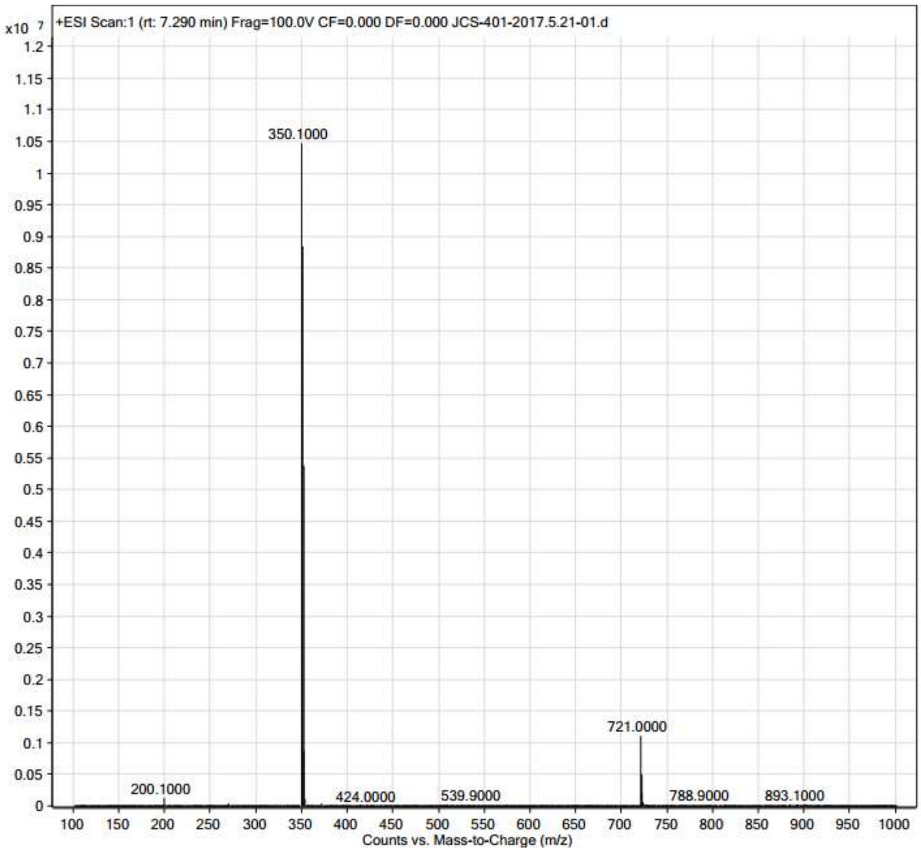

Figure S3 IR spectrum of3

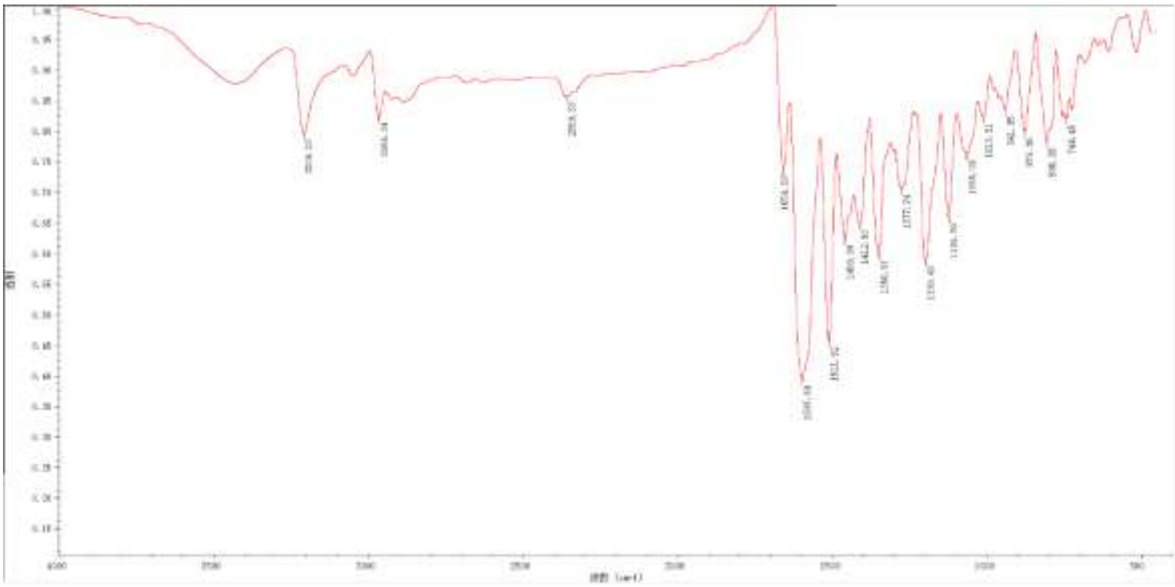

Figure S4 ^{1}H NMR spectrum of ZJ01

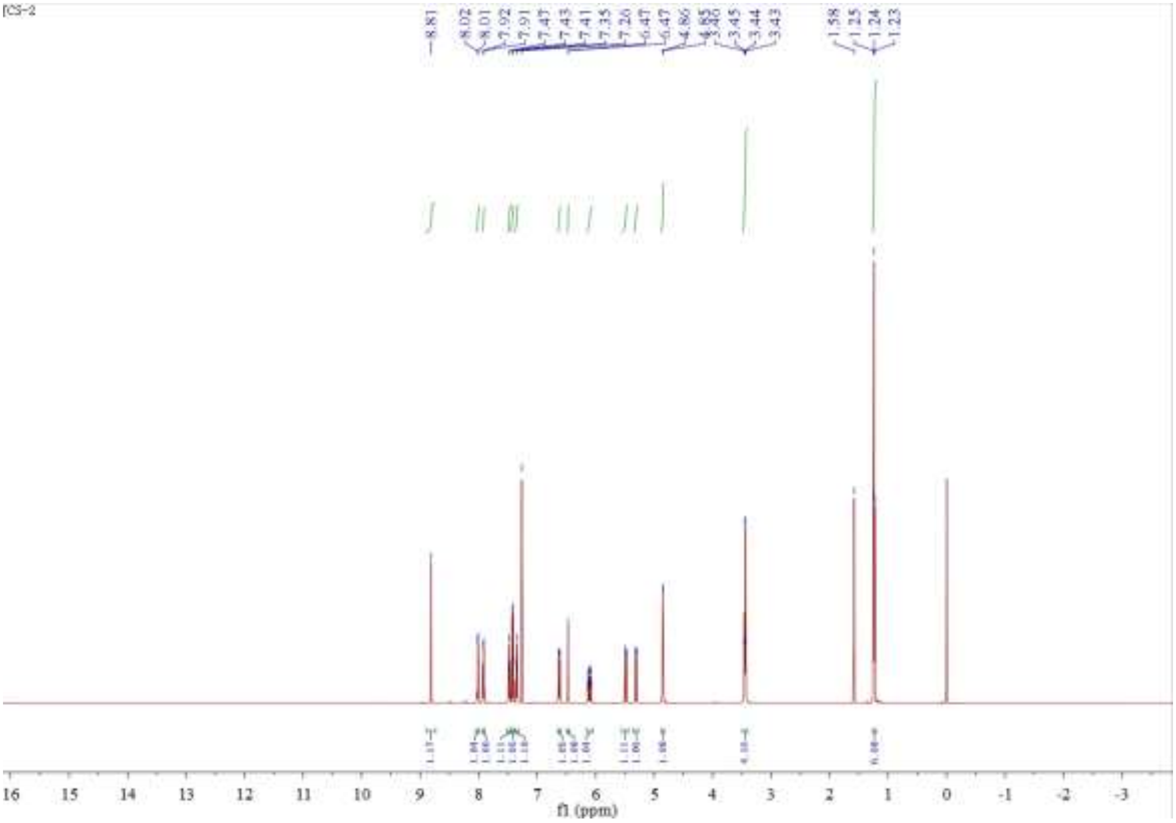

Figure S5 ^{13}C NMR spectrum of ZJ01

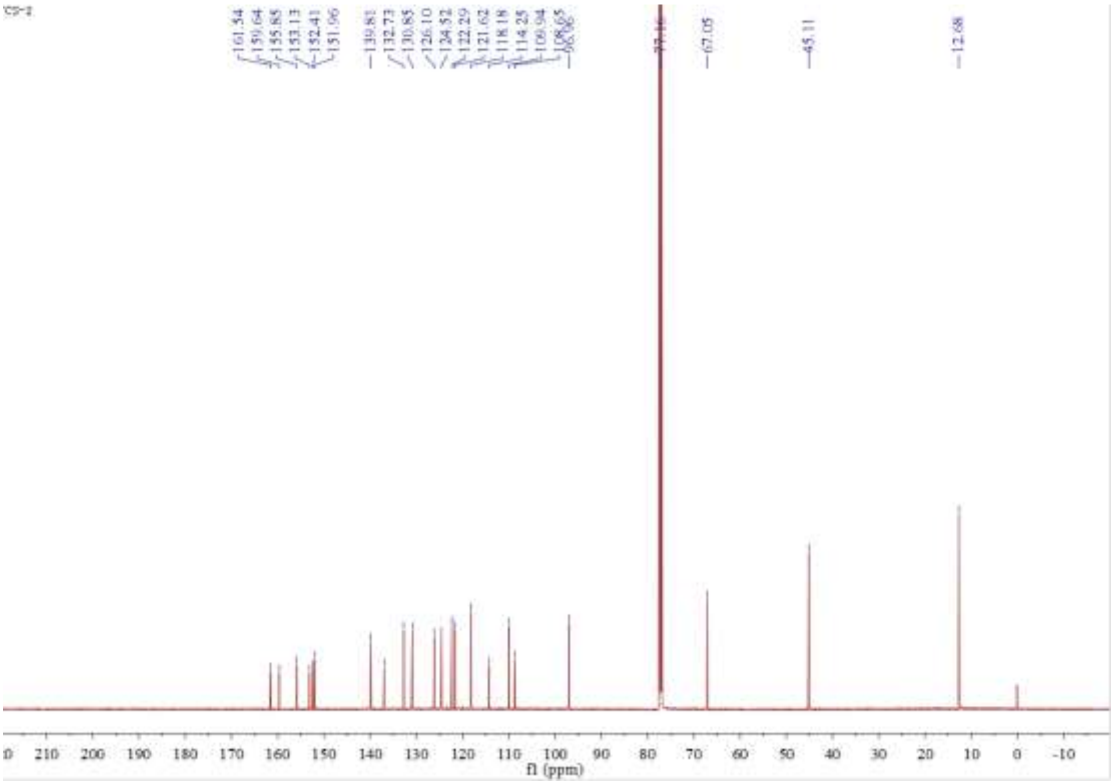

Figure S6 ESIMS spectrum of ZJ01

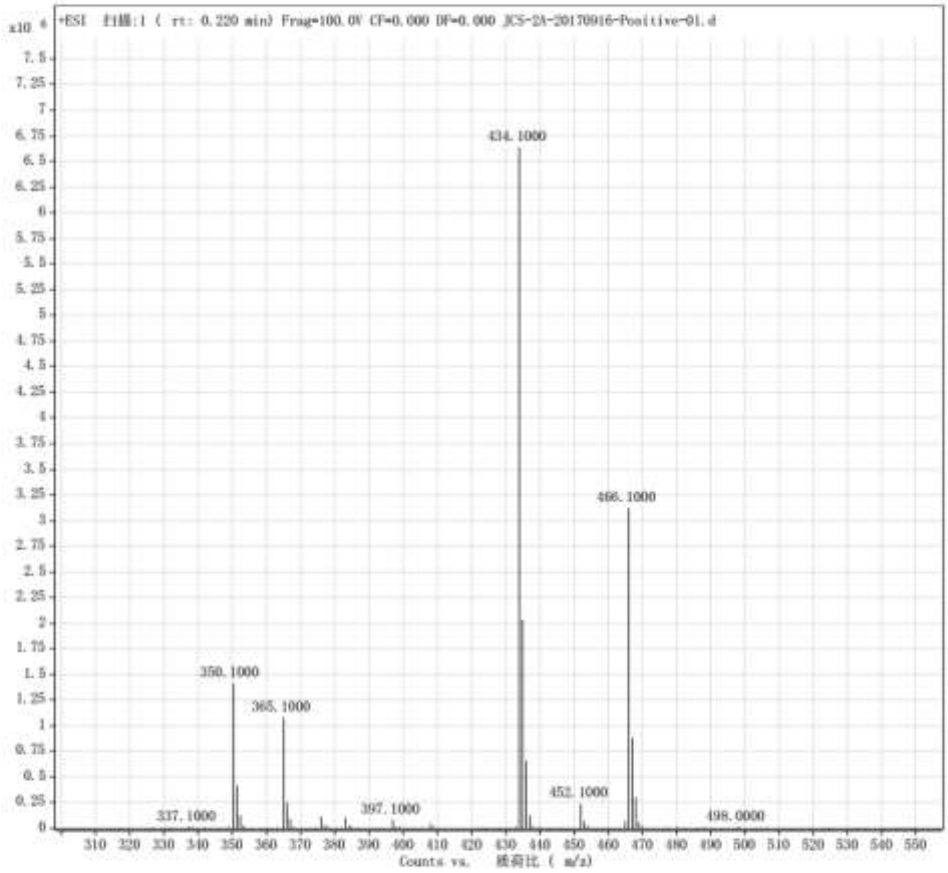

Figure S7 HR-ESIMS spectrum of ZJ01

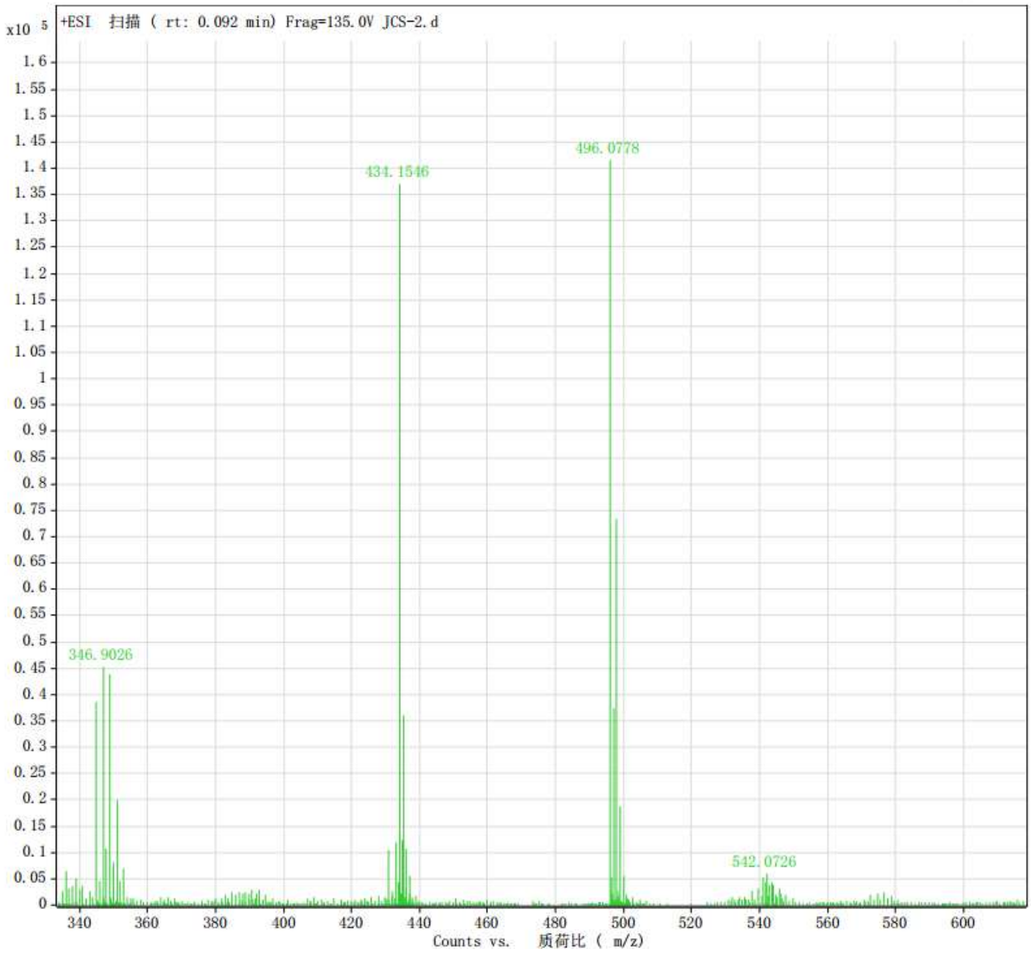

Figure S8 IR spectrum of compound ZJ01

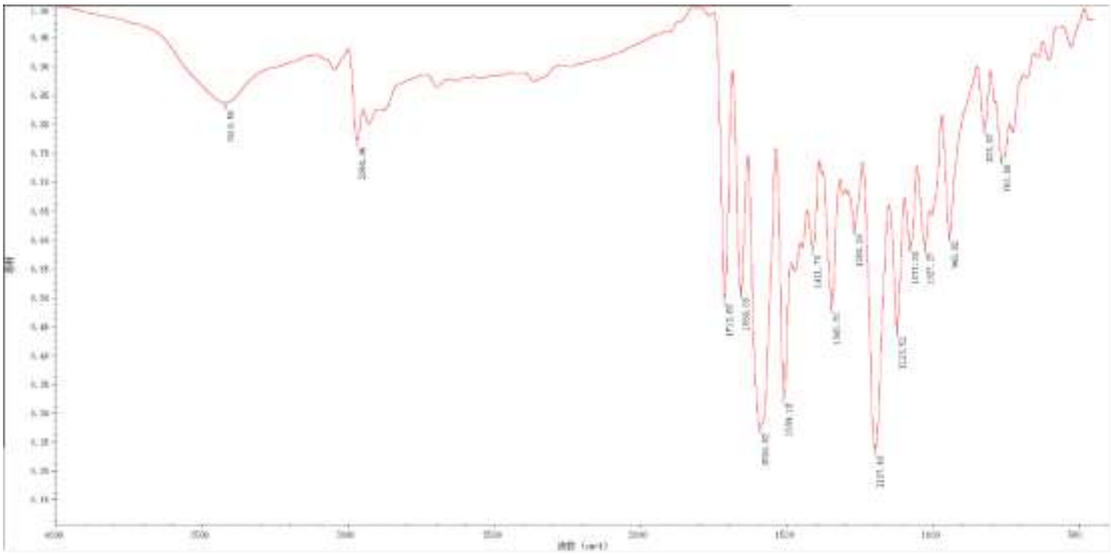

Figure S9 HPLC chromatogram (70–100% MeOH-H_2O in 20 minutes) of ZJ01

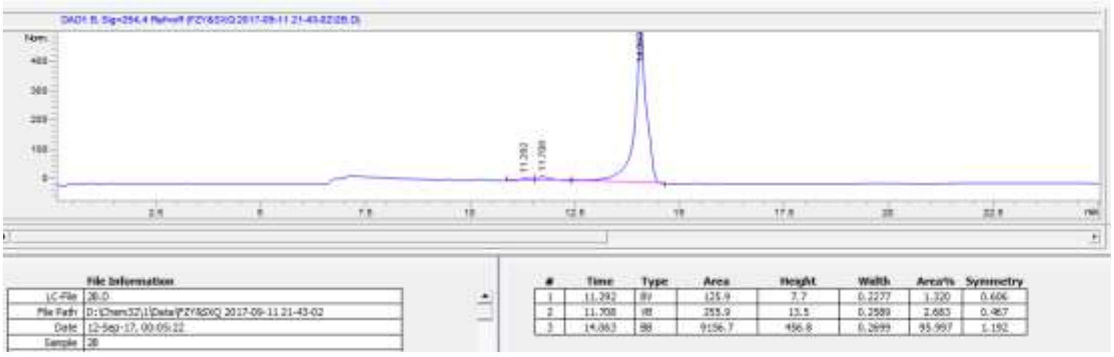

Figure S10 ^{1}H NMR spectrum of ZJ02

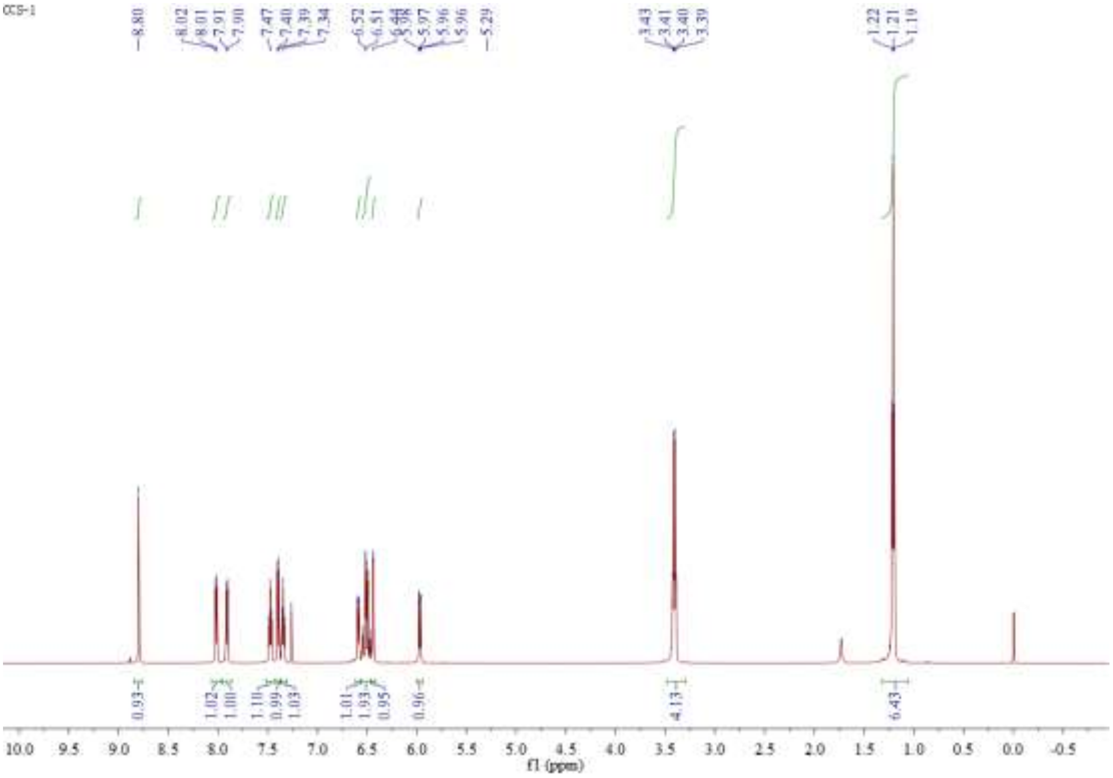

Figure S11 ^{13}C NMR spectrum of ZJ02

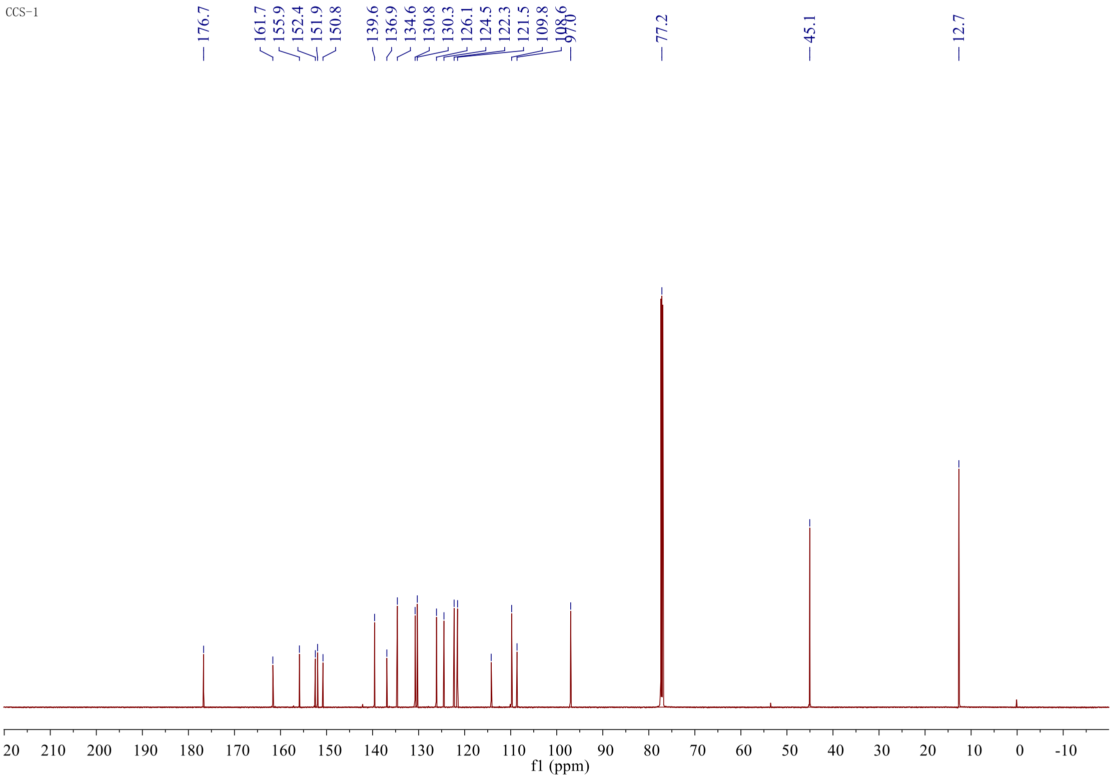

Figure S12 ESIMS spectrum of ZJ02

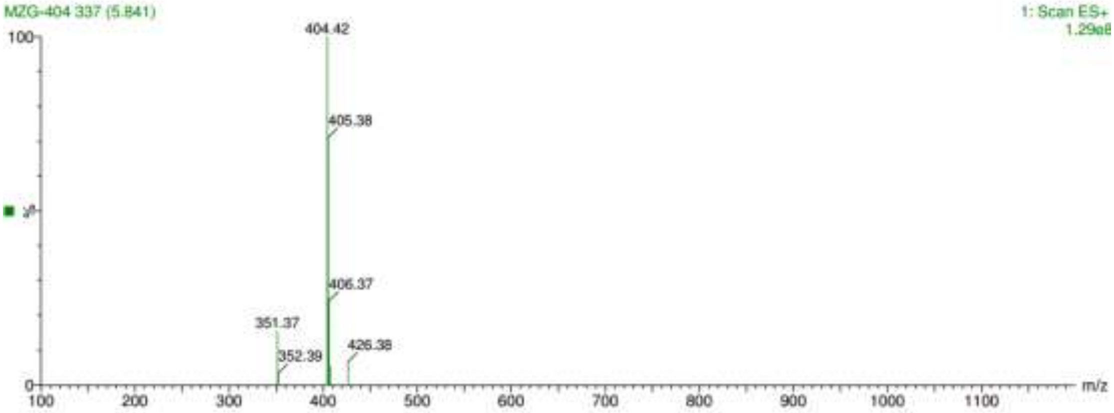

Figure S13　HR-ESIMS spectrum of ZJ02

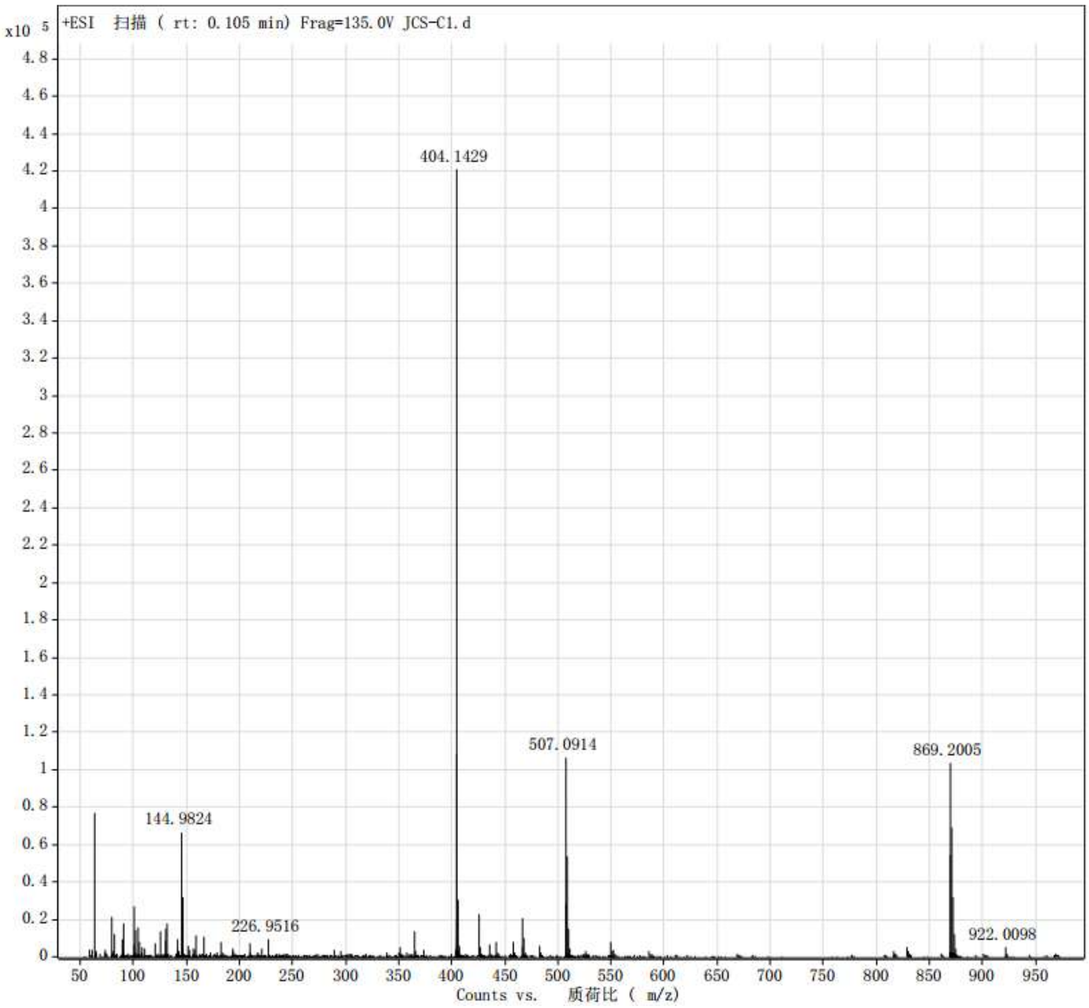

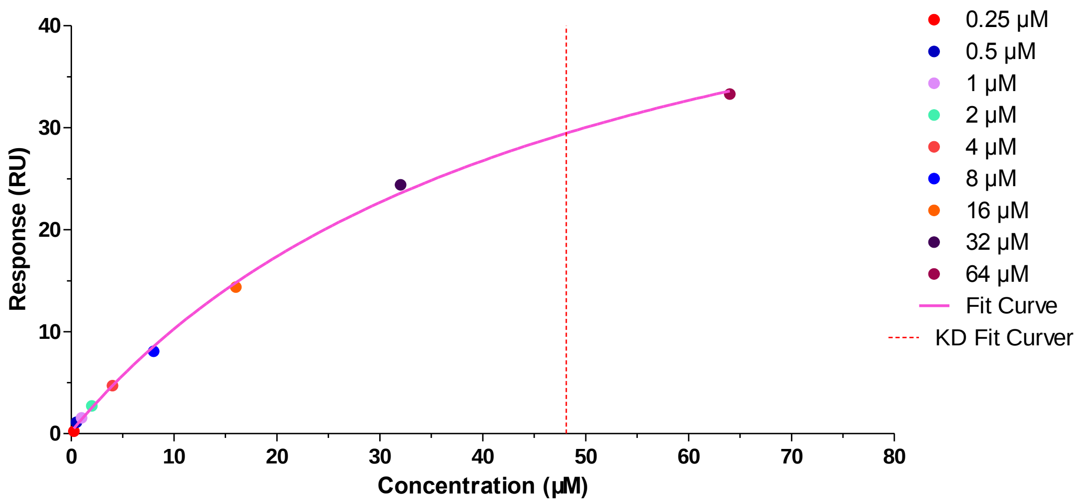

Figure S14 Equilibrium binding curve fit of ZJ01

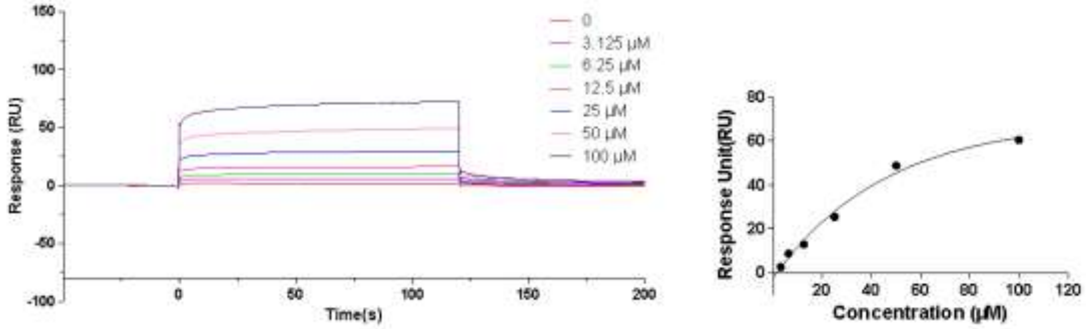

Figure S15 Sensorgrams and equilibrium binding curve fit of S47 in SPR assay

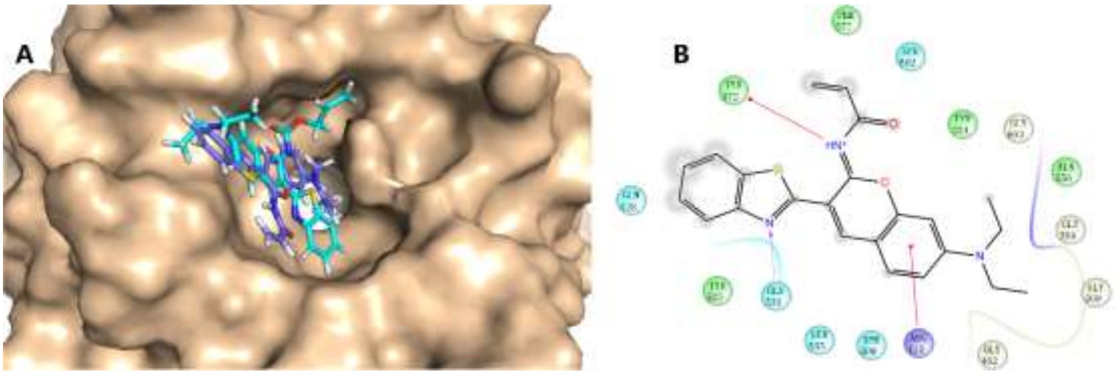

Figure S16 (A) Putative binding mode of **ZJ01** (blue) and **ZJ02** (purple) to Keap1; (B) Schematic diagram showing interactions between **ZJ02** and Keap1.

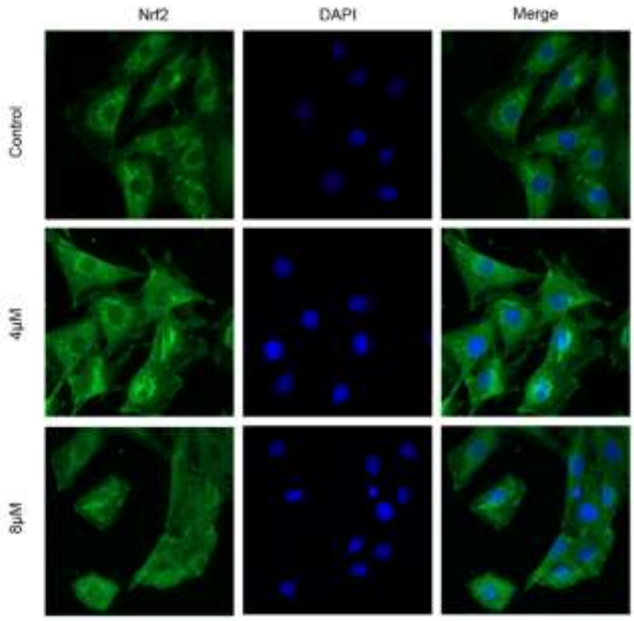

Figure S17 Effect of S47 on the distribution of Nrf2 in H9c2 cells. H9c2 cells were treated with different concentrations of **ZJ01** for 6 h. Immunofluoresence staining analysis of Nrf2 location. Nuclei were counterstained with DAPI.

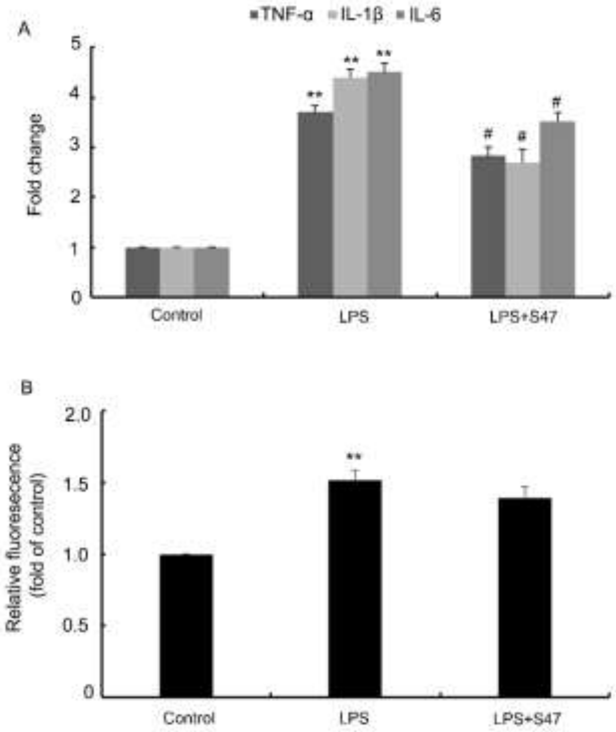

Figure S18 Effects of S47 on the expression of pro-inflammatory cytokines and ROS in LPS-treated H9c2 cells. H9c2 cells were stimulated with 1 μg/ml LPS and treated with or without 8 μM **S47** for 6 h. (A) The expression of pro-inflammatory cytokines TNF-α, IL-1β and IL-6 were determined by RT-PCR; (B) The intracellular ROS level was examined by DCHF. **p < 0.01 vs. control, #p < 0.05 vs. LPS group. n = 3.

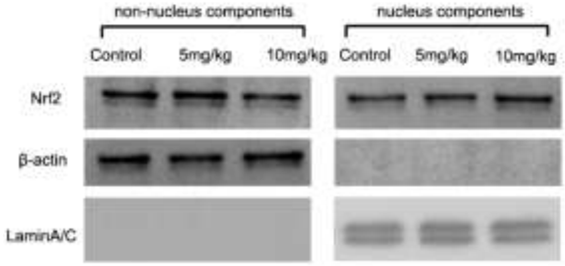

Figure S19 Effect of S47 on the Nrf2 nuclear accumulation *in vivo*. C57BL/6 mice were treated intraperitoneally with different concentrations of **S47** for 12 hours. The nuclear and non-nuclear Nrf2 protein levels of cardiomyocytes were determined by western blotting technology.
